# Bioprospecting of Aerobic Bacteria with Proteolytic Potential Isolated from Animal and Water Sources in the Three Regions of Mainland Ecuador

**DOI:** 10.3390/ijms27062907

**Published:** 2026-03-23

**Authors:** Karla Garcés, Juan Manuel Cevallos, Alisson Sisa, Ana Belén Encalada, Oscar Martínez-Álvarez, Mauricio Mosquera

**Affiliations:** 1 Department of Food Science and Biotechnology, Escuela Politécnica Nacional, Quito P.O. Box 17-01-2759, Ecuador; karla.garces@epn.edu.ec (K.G.); alisson.sisa@epn.edu.ec (A.S.); anabelen.encalada@epn.edu.ec (A.B.E.); 2 Centro de Investigaciones Biotecnológicas del Ecuador (CIBE), Escuela Superior Politécnica del Litoral, Campus Gustavo Galindo Km. 30.5 Vía Perimetral, Guayaquil P.O. Box 09-01-5863, Ecuador; jmceva@espol.edu.ec; 3 Institute of Food Science, Technology and Nutrition (ICTAN-CSIC), 6 José Antonio Novais St., 28040 Madrid, Spain; oscar.martinez@ictan.csic.es

**Keywords:** bioprospecting, protease-producing bacteria, microbial proteases, enzyme production kinetics, *Pseudomonas aeruginosa*

## Abstract

The growing demand for efficient and cost-effective industrial proteases has intensified bioprospecting efforts in diverse ecosystems as a strategy to identify microorganisms with enhanced enzymatic performance. This study aimed to isolate, identify, and evaluate aerobic protease-producing bacteria from animal-protein matrices and water sources collected across the three continental regions of Ecuador, and to assess their suitability for industrial enzyme production A total of 34 bacterial strains were isolated and taxonomically assigned to the orders Enterobacterales, Pseudomonadales, and Bacillales. Proteolytic activity was evaluated using azocasein and casein assays after cultivation in an optimized medium containing 1% soybean paste as an inducer at 37 °C and 120 rpm for 72 h. *Enterobacter cloacae* (BC, pork), *Bacillus paramycoides* (P2, snook), and *Pseudomonas aeruginosa* (CH1, chontacuro) were identified as the most active protease producers from the Andean (Sierra), coastal (Costa), and Amazon regions, respectively. Production kinetics revealed marked strain-dependent differences. BC and P2 reached maximum proteolytic activity on day 4 followed by a decline, whereas CH1 peaked on day 2 and maintained stable activity over time, indicating superior enzymatic stability. Partial purification by gel-filtration chromatography (Sephadex G-100) yielded fractions with enhanced proteolytic activity, while SDS-PAGE analysis confirmed successful enrichment of protease-containing fractions. Overall, the results demonstrate that ecological origin strongly influences protease production and stability, and identify *Pseudomonas aeruginosa* CH1 as a particularly promising candidate for industrial applications requiring robust and sustained proteolytic activity.

## 1. Introduction

Enzymes are essential biocatalysts with a growing global demand, estimated at USD 6.71 billion in 2024 and projected to reach USD 9.84 billion by 2034 [1]. Among them, proteases represent approximately 60% of industrial enzymes marketed and have long been of interest to the detergent, textile, food, cosmetic, and pharmaceutical industries [2]. Recent advances in biotechnology, together with the drive for eco-friendly, cleaner alternatives to conventional chemicals, have broadened their applications to protein modification, waste treatment, and the production of bioactive peptides, among others [3,4].

Proteases are essential biological catalysts that hydrolyze peptide bonds in proteins, generating shorter peptide chains [5]. According to the Enzyme Commission classification, proteases fall under class 3 (hydrolases), subclass 4, and each proteolytic enzyme is assigned a unique identifier in the format EC 3.4.x.x. These enzymes are categorized based on several parameters, including their site of action, substrate specificity, optimal pH range, and catalytic mechanism, which involves a specific amino acid residue located in the active site. Depending on their site of action, proteases are classified as endopeptidases (which hydrolyze non-terminal peptide bonds) or exopeptidases (which hydrolyze peptide bonds at the terminal ends of the substrate) [2].

These enzymes can be obtained from plants, animals, and microbial sources, with the latter being the most widely used worldwide, accounting for approximately 45% of the protease market [3]. This preference is due to their notable advantages, such as high production rates, stability, specificity, and independence from climatic conditions or ethical constraints. An additional significant advantage of microbial sources is the feasibility of applying enzyme and genetic engineering approaches, as well as molecular and computational biology tools, to generate improved strains that enable more efficient enzyme production due to notable advantages such as high growth rates, stability, specificity, and the amenability to improvement via genetic engineering and molecular biology [6,7].

Although numerous protease-producing bacteria have been identified, many are unable to withstand drastic environmental changes and, under industrial conditions, fail to optimize their parameters to achieve optimal yields. Consequently, they are unable to produce the quantities required to meet industrial demand [8]. Consequently, microorganisms employed for protease production are typically isolated from environments with specific conditions that mirror the desired functional traits of the target enzyme [9]. For example, microorganisms with desirable enzymatic properties are often isolated from environments rich in proteinaceous residues such as food-processing wastes, hot springs, poultry or meat by-products, and tannery effluents. These niches possess distinctive physicochemical conditions that select for bacteria capable of producing proteases adapted to extreme pH, temperature, and substrate availability, making them valuable sources of enzymes suitable for industrial applications [2].

Efficient production of proteolytic enzymes depends on the use of appropriate culture media and specific conditions tailored to the requirements of each microorganism. Two main fermentation methods are employed for protease production: solid-state fermentation (SSF) and submerged fermentation (SmF), the latter being the most widely used in industry, accounting for approximately 90% of industrial processes. The preference for submerged fermentation (SmF) is attributed to the ease of controlling physicochemical variables, improved distribution of medium components, and more efficient recovery of extracellular enzymes [2,10].

Another relevant factor is that substrates used for enzyme production must provide highly bioavailable nutrients at low cost. In this regard, the utilization of agro-industrial by-products represents an ideal alternative, as they are generated in large quantities and pose significant environmental challenges [11]. One such by-product is soybean meal, a protein-rich residue obtained from the oil extraction industry, which has proven to be an ideal substrate for protease production at the laboratory scale, with high potential for industrial-scale implementation due to its high yield and low cost [12].

Once proteolytic enzymes are obtained, purification processes are essential and involve multiple methods that must be carefully selected to achieve high-quality enzymes at low cost. The recommended initial step is enzyme concentration using organic solvents (methanol, ethanol, isopropanol, or acetone) or ammonium sulfate precipitation [2]. Subsequently, chromatographic techniques such as gel filtration chromatography are employed. This method uses a molecular exclusion resin (e.g., Sephadex G-100) to separate proteins, including proteases, based on their molecular size while preserving their structural and functional integrity [13].

Regarding the quantification of proteolytic activity in the resulting enzyme concentrates, several methods are available, with colorimetric and spectrophotometric assays being widely used due to their accuracy and ease of standardization. Among these, two commonly employed methods use casein and azocasein as substrates, which are hydrolyzed by proteases, followed by spectrophotometric measurement of the released products [14,15].

Based on the above, this study focuses on characterizing bacterial diversity and assessing the industrial potential for enzyme production of strains isolated from animal-protein samples and water sources collected in three cities across different climatic regions of continental Ecuador. The goal is to know the best region and source of proteolytic enzymes with the lowest substrate specificity, and the highest stability and efficiency.

## 2. Results and Discussion

### 2.1. Bacterial Bioprospecting

A total of 34 bacterial strains were isolated from food matrices and water sources collected in three cities representing different climatic regions of continental Ecuador, as detailed in Table 1. This distribution reflects the wide microbial diversity of the sampled environments and suggests the combined influence of local climate and matrix composition on bacterial isolation.

Previous reports highlight the wide distribution of protease-producing microorganisms in diverse environments. For instance, *Bacillus subtilis* B13 was isolated from beef and shown to produce collagenolytic proteases with an optimal pH of 7.5 and a temperature below 50 °C, making it suitable for use as a meat tenderizer [16].

Similarly, *Staphylococcus simulans* QB7 was isolated from fermented meat products, where it promoted the degradation of proteins and lipids [17]. However, there is also a high likelihood of isolating pathogenic bacteria such as *Clostridium*, *Vibrio*, or *Proteus*, which can produce collagenolytic enzymes but whose toxin production limits their application in the food and pharmaceutical industries. Therefore, evaluating virulence genes associated with foodborne intoxication risk is essential [16,17].

A *Bacillus cereus* strain isolated from the intestine of the fish *Mugil cephalus* has been reported to produce halotolerant proteases with potential applications in aquafeed [18]. Likewise, *Bacillus proteolyticus* CFR3001, isolated from fish-processing waste, produces an alkaline protease with antibacterial properties capable of inhibiting pathogenic bacteria such as *Escherichia coli* and *Listeria monocytogenes* [19].

With respect to chontacuro, (*Rhynchophorus palmarum* L.), no prior reports describing the isolation of proteolytic bacteria were found. Nevertheless, it is considered in the present study because it is a traditional, representative food of the Amazon region and is characterized by an excellent nutritional profile and high biological value [20].

Water sources such as rivers or fish ponds have been reported to be dominated by *Proteobacteria*, *Cyanobacteria*, *Bacteroidetes*, and *Actinobacteria*, groups with the potential to produce hydrolytic enzymes due to the abundance of organic matter rich in proteins derived from food residues, scales, or excreta. For instance, *Exiguobacterium indicum* was isolated from an aquaculture system, exhibiting high proteolytic activity and potential for use in aquatic bioremediation systems [21]. Bacterial sampling from biofilms was also considered, since these structures harbor bacterial communities embedded within a matrix of polymeric substances, which confers greater resistance to environmental stress factors [22].

### 2.2. Bacterial Identification

#### 2.2.1. Biochemical Tests

The biochemical tests detailed in Table A1 allowed for the inference of active metabolic pathways in the isolated bacterial strains, contributing to their presumptive identification through comparison with known metabolic profiles.

Gram staining revealed a predominance of Gram-negative bacteria (31 strains), while three strains were identified as Gram-positive, two of which exhibited spore-forming structures, a morphological trait characteristic of the genera *Bacillus* or *Paenibacillus*, both frequently associated with nutrient-rich environments and known for their ability to secrete extracellular proteases [23]. All strains showed positive catalase activity, confirming the predominance of aerobic or facultative anaerobic organisms [14]. Urease activity was detected in approximately 70% of the isolates, mainly those obtained from meat matrices and aquatic biofilms, suggesting adaptations to nitrogen metabolism [24].

The tests performed on Triple Sugar Iron (TSI) agar allowed for differentiation of bacterial strains according to their ability to ferment carbohydrates and to produce hydrogen sulfide (H_2_S) and gas. Among the 34 bacterial strains, 22 were able to ferment glucose, lactose, and/or sucrose; five fermented only glucose; and seven were non-fermenters of carbohydrates. Gas and H_2_S production varied among isolates, providing additional evidence of specific fermentative pathways [25].

Growth of 31 isolates on MacConkey agar confirms their enteric Gram-negative nature, and 26 of these were lactose fermenters. Three strains did not grow on this medium, which is consistent with Gram-positive or non-enteric bacteria. Bacterial motility was positive in 18 isolates, indicating the possible presence of peritrichous or polar flagella, a feature common in genera such as *Pseudomonas*, *Bacillus*, and *Enterobacter* [26].

Finally, the gelatin liquefaction test, associated with the production of extracellular proteolytic enzymes (gelatinases), was positive in seven strains distributed across the Coastal and Amazon regions [27].

Overall, the phenotypic profile is consistent with bacteria belonging to the orders *Enterobacterales* (e.g., *Escherichia*, *Klebsiella*, *Enterobacter*, *Citrobacter*), *Pseudomonadales* (e.g., *Pseudomonas*, *Acinetobacter*), and *Bacillales* (e.g., *Bacillus*, *Paenibacillus*).

#### 2.2.2. Molecular Identification

**Figure 1:**
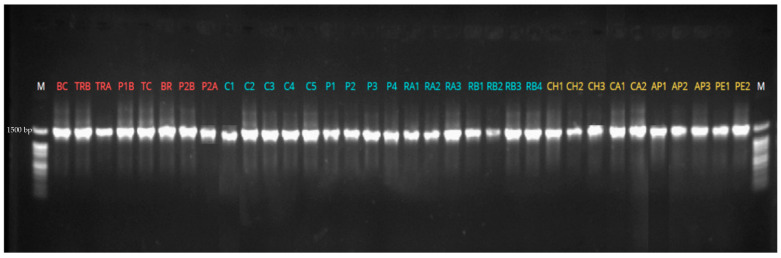
Electrophoretic profile of PCR-amplified 16S rRNA gene products from isolated bacterial strains. The lanes highlighted in red, light blue, and yellow represent the amplified products of bacterial strains isolated from cities located in the Andean, Coastal, and Amazon regions respectively.

Molecular identification was performed by PCR amplification of the 16S rRNA gene followed by Sanger sequencing. The PCR products (amplicons) were analyzed by electrophoresis on 1% agarose gel to verify their size and integrity. The electrophoretic profile is shown in Figure 1, where lane “M” corresponds to the 100 bp molecular weight marker (BioBasic, Ready-to-use, Markham, ON, Canada), and the lanes highlighted in red, light blue, and yellow represent the amplified products of bacterial strains isolated from cities located in the Andean, Coastal, and Amazon regions, respectively. A single band of approximately 1500 bp was observed in all lanes, confirming the specificity of the amplification and the suitability of the products for Sanger sequencing [28].

For taxonomic assignment, the sequences obtained were compared using BLASTn against the GenBank (NCBI) database, considering alignments valid when the E-value was 0.0 and the sequence identity was ≥98%. The species showing the highest similarity are listed in Table 2 [29]. However, it should be noted that for certain known species complexes—*Enterobacter cloacae* complex, *Klebsiella pneumoniae* complex, *Bacillus cereus sensu lato*, and the *Escherichia/Shigella* clade—the resolution of the 16S rRNA gene is limited. Complementary molecular markers such as gyrB, rpoB, and recA, as well as whole-genome sequencing (WGS), provide higher resolution and reliability for species and subspecies identification. Therefore, these identifications are considered tentative, based on their correlation with the biochemical tests described in the previous section [30].

### 2.3. Evaluation of the Proteolytic Capacity of the Identified Strains

#### 2.3.1. Enzymatic Activity Quantification

**Figure 2:**
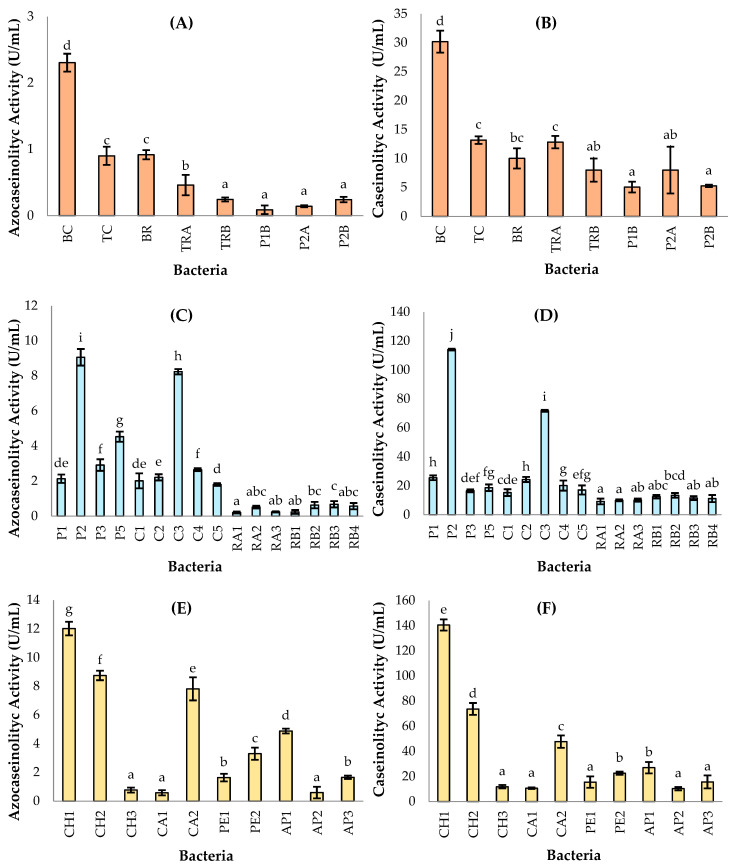
Evaluation of proteolytic activity of bacterial isolates from: (**A**,**B**) Andean region; (**C**,**D**) Coastal region; (**E**,**F**) Amazon region. Means sharing a common letter are not significantly different (*p* > 0.05).

Figure 2 shows the results of proteolytic activity quantification for the bacterial strains using both azocasein and casein hydrolysis assays. The letters assigned by Duncan’s test (α = 0.05) confirmed statistically significant differences among the evaluated bacterial isolates.

The strains exhibiting the highest proteolytic enzyme production corresponded to *Pseudomonas aeruginosa* (CH1—chontacuro), *Bacillus paramycoides* (P2—snook), *Serratia marcescens* (CH2—chontacuro), and *Pseudomonas paraeruginosa* (C3—pork), which clustered at the upper levels and differed significantly from the low to moderate profiles observed in isolates from the Andean region and aquatic sources.

Several studies have used gelatin hydrolysis as a screening test to detect protease-producing bacteria; however, its limited sensitivity and specificity may lead to false positives or negatives. Therefore, quantitative evaluation provides more reliable and reproducible results [31].

Regarding the strains isolated from the Andean region, none exhibited positive gelatin hydrolysis or high quantitative activity values. However, *Enterobacter cloacae* (BC), isolated from pork, showed the highest activity within this group, with values of 2.31 ± 0.14 U/mL and 30.20 ± 1.90 U/mL for azocasein and casein, respectively. These values could potentially be optimized by using mineral media enriched with specific inducers of protease secretion. In this context, Fajingbesi et al. [32] reported that the inoculation of *E. cloacae* in a mineral medium adjusted to pH 9, supplemented with fish viscera, and incubated for 72 h at 35 °C and 140 rpm, resulted in a significantly higher protease yield. However, this remains, to date, the only study reporting protease identification from this bacterial strain, which has been primarily characterized industrially for its ability to produce cellulases and dehydrogenases.

In the Coastal and Amazon regions, seven isolates exhibited positive gelatin hydrolysis, indicative of extracellular protease secretion. Among them, P1 and P2 were identified by 16S rRNA gene BLAST analysis as *Bacillus paramycoides* (100% identity), a microorganism previously reported to produce thermostable and alkaline enzymes [33]. However, their biochemical profiles differed in urease production and carbohydrate fermentation and showed marked contrasts in proteolytic activity: for azocasein and casein, respectively, P1 exhibited 2.14 ± 0.47 and 25.57 ± 0.60 U/mL, whereas P2 reached 9.07 ± 0.33 and 114.13 ± 1.02 U/mL. These discrepancies support the hypothesis that they represent distinct strains with high similarity in conserved genes—such as 16S rRNA, which has limited resolution for intraspecific discrimination and within the *Bacillus cereus* sensu lato complex—but with divergences in gene content and/or regulation of protease expression and secretion pathways [34].

Similarly, isolates P3 and CH1 hydrolyzed gelatin and were identified by 16S rRNA gene BLAST analysis as *Pseudomonas aeruginosa*, a bacterium well known for its ability to secrete alkaline proteases, elastases, and metalloproteases, which contribute to its robustness and versatility [35]. However, they differed in motility: P3 showed no displacement on semisolid agar, whereas CH1 was motile. Loss of motility in *P. aeruginosa* may result from mutations in structural flagellar genes or in components of type IV pili, which can influence its proteolytic capacity. Indeed, proteolytic activities against azocasein and casein (U/mL) were 2.92 ± 0.29 and 16.58 ± 2.27 for P3, compared to 12.02 ± 0.47 and 140.53 ± 4.45 for CH1, respectively [36].

The isolate C3 was identified as *Pseudomonas paraeruginosa*, a species recently segregated from the atypical *P. aeruginosa* clade and proposed as a new taxon in 2022. Owing to its recent classification, studies on its proteolytic capacity are scarce; nevertheless, its close phylogenomic relationship to *P. aeruginosa* suggests similar enzymatic mechanisms. Consistent with this, isolate C3 showed a positive gelatinase reaction and activities of 8.24 ± 0.08 U/mL (azocasein) and 71.73 ± 3.48 U/mL (casein) under the tested conditions [37].

The strains C5 and CH2 were identified as *Serratia marcescens*, a species known to secrete extracellular metalloproteases of the serralysin family, such as serratiopeptidase, which exhibits anti-inflammatory and fibrinolytic properties of pharmaceutical interest. Both isolates hydrolyzed gelatin; however, CH2 showed substantially higher proteolytic activity than C5 for both substrates, with the following values: CH2 (8.75 ± 0.33 and 73.67 ± 4.74 U/mL) and C5 (1.80 ± 0.06 and 17.10 ± 1.95 U/mL), highlighting phenotypic differences in enzyme production even within the same taxon. Conversely, strain C2, also identified as *S. marcescens*, did not hydrolyze gelatin but exhibited measurable activity against azocasein and casein (2.22 ± 0.15 and 24.37 ± 0.71 U/mL, respectively), suggesting possible regulation dependent on culture conditions or a false negative in the qualitative assay [38,39].

Taken together, these results indicate that the ecological origin and the availability of proteinaceous substrates in the environment influence the selection and expression of phenotypes with greater proteolytic capacity. Accordingly, strains BC, P2, and CH1 were identified as the highest protease producers from the Andean, Coastal, and Amazon regions, respectively.

#### 2.3.2. Enzyme Production and Bacterial Growth Kinetics

The temporal patterns of protease production observed in this study are consistent with previous reports indicating that bacterial protease synthesis is closely linked to specific phases of microbial growth. In several bacterial species, protease secretion typically initiates during the exponential phase and reaches maximum levels in the late logarithmic or early stationary phase, coinciding with nutrient depletion and the need to hydrolyze complex substrates to sustain metabolism. Similar behavior has been reported for *Aeromonas* spp., where maximum protease activity was detected between 18 and 20 h of incubation, followed by a gradual decline despite residual enzymatic activity persisting up to 72 h, highlighting a proportional relationship between cell growth and enzyme production [40].

In the present study, once the bacteria with the highest proteolytic output had been identified, the kinetics of enzyme production were evaluated over seven days of culture to determine the day of maximum production and to characterize the microbial growth pattern. For each isolate, one-way ANOVA was applied, with incubation day as the factor and enzyme activity as the response variable, measured by azocasein and casein hydrolysis in separate models.

**Figure 3:**
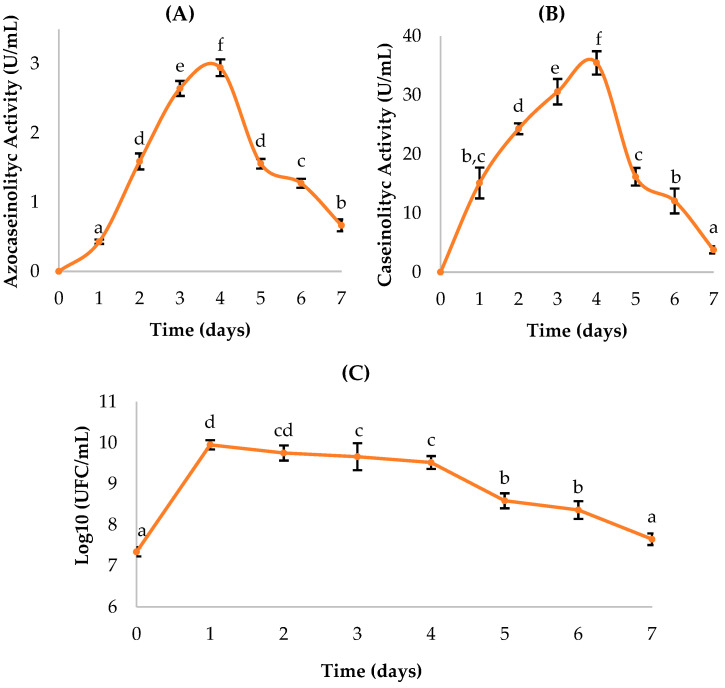
Kinetics of *Enterobacter cloacae* (BC): (**A**,**B**) proteolytic activity quantification; (**C**) bacterial growth. Means sharing a common letter are not significantly different (*p* > 0.05).

The *Enterobacter cloacae* (BC) strain showed a clear temporal variation in proteolytic enzyme production and cell growth (Figure 3). Enzymatic activity determined with azocasein increased progressively from day 1 (0.43 ± 0.03 U/mL) to a maximum on day 4 (2.95 ± 0.12 U/mL), followed by a gradual decline in subsequent days. A similar pattern was observed using the casein assay, with maximum activity also occurring on day 4 (35.47 ± 1.98 U/mL), suggesting that enzyme production is coupled to the active growth phase.

The bacterial count, expressed as log_10_ CFU/mL, peaked on day 1 (9.95 ± 0.11), indicating an early logarithmic phase, followed by a progressive decrease from day 2 onward, coinciding with a reduction in cell density but not necessarily in enzymatic activity.

This suggests that the enzyme is extracellular in nature and remains active in the medium even as bacterial growth declines. Statistical analyses using ANOVA and Duncan’s test (*p* < 0.05) confirmed significant differences among days for both proteolytic activity and bacterial growth, with day 4 being statistically superior in both enzymatic detection methods (letter “f”). These findings differ from those reported by Fajingbesi et al. [32] in which the peak of protease production by *E. cloacae* occurred at 72 h (third day) of incubation under optimal conditions.

**Figure 4:**
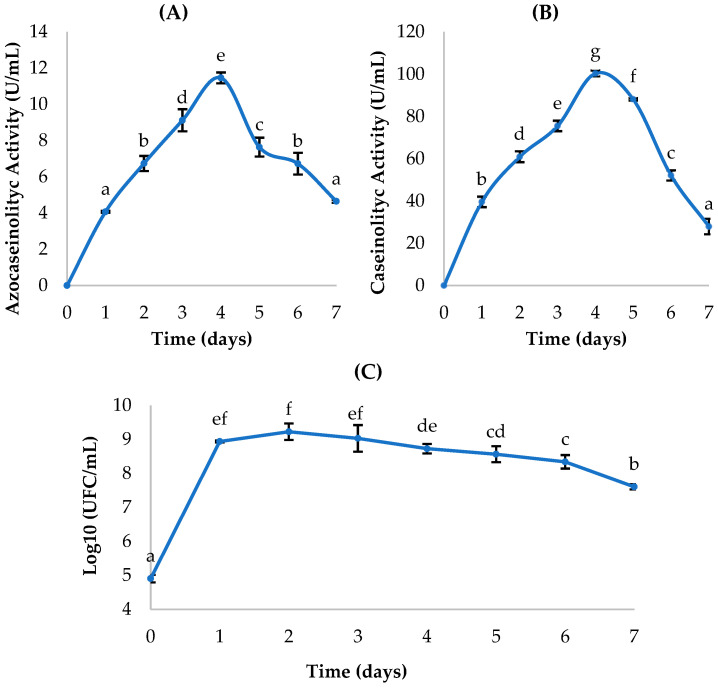
Kinetics of *Bacillus paramycoides* (P2): (**A**,**B**) proteolytic activity quantification; (**C**) bacterial growth. Means sharing a common letter are not significantly different (*p* > 0.05).

*Bacillus paramycoides* (P2) exhibited maximum proteolytic activity on day 4, with values of 11.46 ± 0.30 U/mL for azocasein and 100.35 ± 1.40 U/mL for casein. From this point onward, a gradual decrease in activity was observed, possibly associated with enzyme degradation or suboptimal conditions for its expression (Figure 4). In contrast, bacterial growth reached its peak on day 2, with approximately 9.22 ± 0.24 log_10_ CFU/mL, corresponding to the exponential phase. Subsequently, a progressive decline in cell concentration was recorded, marking the onset of the stationary and death phases.

The time lag observed between maximum cell growth and peak enzymatic production suggests that protease synthesis is not strictly associated with bacterial growth but follows a non-growth-dependent pattern, typical of secondary metabolites. This behavior is relevant for determining the optimal time for collecting the enzymatic extract, which in this case corresponds to the fourth day of culture, when the highest concentrations of active protease are achieved.

These results partially differ from those reported by Gat et al. [33], who cultivated a *B. paramycoides* strain in medium supplemented with 3% potato peel and observed maximum proteolytic activity at 72 h under alkaline conditions (pH 9) at 40 °C. Additionally, in an optimization approach, Alshehri et al. [41] applied response surface methodology (RSM) and achieved peak production at 8 h, at pH 7 and 45 °C; they also characterized the protease as thermostable, alkaline, and detergent compatible. Collectively, these studies indicate that optimizing culture conditions is essential to reveal the full proteolytic potential of this bacterium.

**Figure 5:**
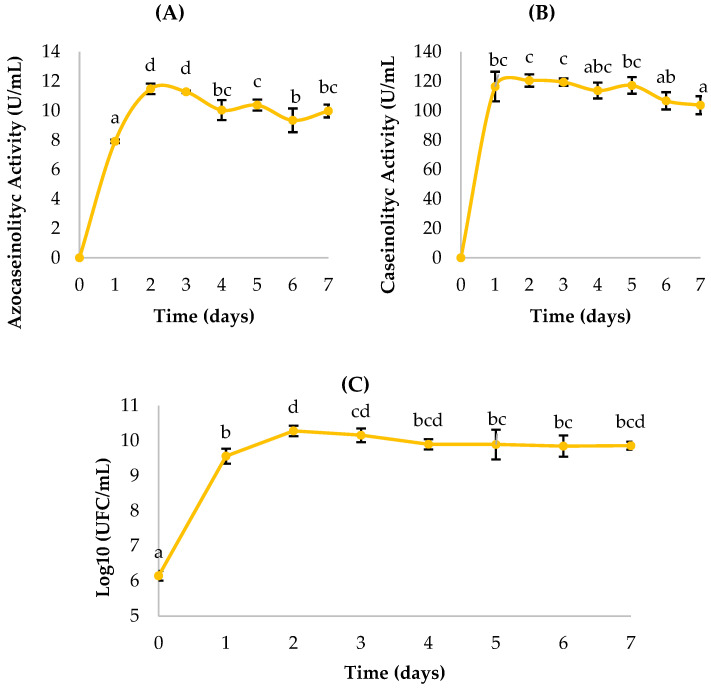
Kinetics of *Pseudomonas aeruginosa* (CH1): (**A**,**B**) proteolytic activity quantification; (**C**) bacterial growth. Means sharing a common letter are not significantly different (*p* > 0.05).

Figure 5 shows the kinetics of *Pseudomonas aeruginosa* (CH1), which exhibited a remarkable proteolytic capacity over time, as evidenced by the consistently high enzymatic activity values obtained with both assay methods. Azocasein activity increased from day 1 (7.92 ± 0.11 U/mL) to a maximum on day 2 (11.48 ± 0.36 U/mL), similar to the casein assay, where values reached 116.43 ± 10.04 and 120.50 ± 4.19 U/mL on days 1 and 2, respectively.

The observed differences were statistically significant according to ANOVA and Duncan’s test (*p* < 0.05). Day 2 was identified as the point of maximum proteolytic production; however, it did not differ significantly from day 3, indicating that the enzymatic extract could be collected on either day. Regarding bacterial growth, the logarithmic count reached its peak on day 2 (10.28 ± 0.15) and remained high until day 7.

This pattern differs from that reported by Zambare et al. [42], in which maximum protease production was achieved after 72 h of incubation under optimized conditions, with an initial production phase associated with cell growth up to 48 h, followed by a biomass decline phase coinciding with a sustained increase in enzyme secretion.

Consistently, in a feather biodegradation study, *P. aeruginosa* was reported to produce high levels of keratinases after 48 h of fermentation in a feather-based medium at 40 °C and pH 8.0, achieving a degradation efficiency of 95%. This behavior resembles the protease obtained in the present study, which exhibited sustained proteolytic stability over time [43], suggesting that the enzyme is extracellular in nature, with production coupled to the late logarithmic phase and catalytic stability during the stationary phase. Overall, the combination of high biomass and elevated enzyme production positions this strain as a promising candidate for biotechnological applications requiring high protease yield and stability.

#### 2.3.3. Partial Enzymatic Purification

During the enzyme partial purification stage, extracts collected on the day of maximum production from *Enterobacter cloacae* (BC), *Bacillus paramycoides* (P2), and *Pseudomonas aeruginosa* (CH1) were subjected to gel filtration chromatography using Sephadex G-100, and their proteolytic activity (U/mL) was determined using azocasein and casein as substrates.

**Figure 6:**
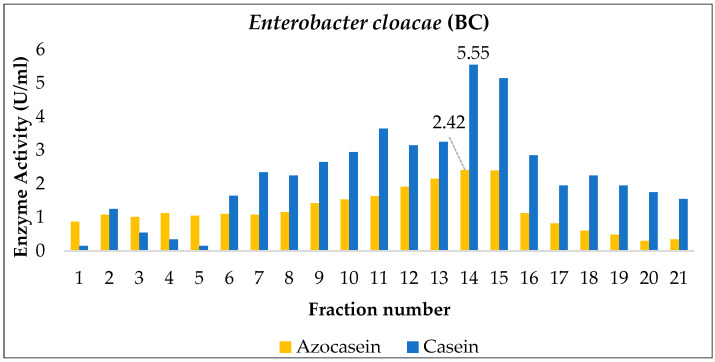
Partial purification of the enzyme produced by *Enterobacter cloacae* (BC) via Sephadex G-100 gel filtration.

**Figure 7:**
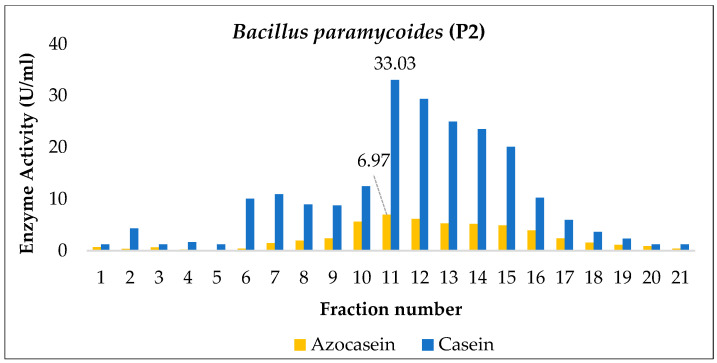
Partial purification of the enzyme produced by *Bacillus paramycoides* (P2) via Sephadex G-100 gel filtration.

The *Enterobacter cloacae* (BC) and *Bacillus paramycoides* (P2) strains showed maximum activity in fractions 14 and 11, respectively (Figure 6 and Figure 7). In both cases, no increase in enzymatic activity was observed compared to the crude extract. However, it is important to note that chromatographic efficiency should not be evaluated solely based on enzymatic activity (U/mL), since this value tends to be higher in the crude extract due to the presence of non-target proteins and decreases at each purification step because of protease dilution. Therefore, the specific activity (U/mg), which relates enzymatic units to total protein content, should be quantified to estimate the purification factor, which typically increases progressively throughout the purification process [44].

For instance, in the alkaline protease produced by *Bacillus cereus* AUST-7 [13], the total enzymatic activity decreased after Sephadex G-100 chromatography due to inherent process losses (adsorption to the matrix, mechanical losses, or partial inactivation). Nevertheless, the specific activity increased from 260.02 U/mg in the crude extract to 8902 U/mg, representing a purification factor of 35.91. This pattern—characterized by a reduction in total activity but a marked increase in specific activity—is typical of enzymatic purification processes, where the main goal is to obtain a preparation enriched in the enzyme of interest, even at the expense of lower overall yield [44].

A comparable behavior has been reported for proteases produced by other *Bacillus* species. In *Bacillus siamensis* CSB55, protease production initiated at 16 h and reached its maximum at 64 h of cultivation at 37 °C, followed by a three-step purification strategy involving ammonium sulfate precipitation (40–80%) and two size-exclusion chromatography steps using Sepharose CL-6B and Sephadex G-75. Despite the relatively low final yield (16.23%), the purified enzyme (SH21) exhibited a high specific activity of 2926.67 U/mg and achieved a 23.09-fold purification. These results clearly illustrate that multistep purification schemes often lead to substantial losses in total enzymatic activity while significantly increasing enzyme purity, supporting the interpretation that the lack of increased U/mL observed after gel filtration in the present study does not necessarily indicate poor purification efficiency but rather reflects the inherent trade-off between yield and purity in protease purification processes [45].

**Figure 8:**
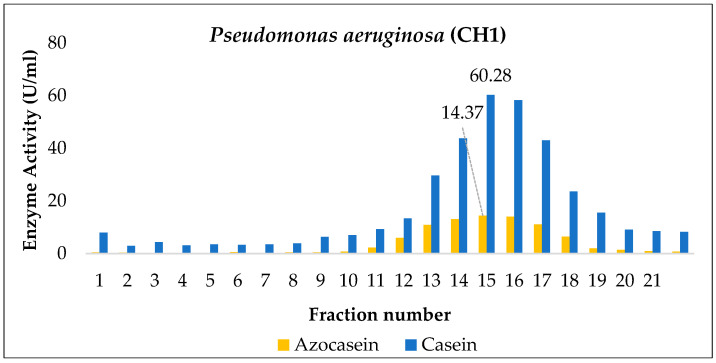
Partial purification of the enzyme produced by *Pseudomonas aeruginosa* (CH1) via Sephadex G-100 gel filtration.

Regarding the elution profile of *Pseudomonas aeruginosa* (CH1), the maximum proteolytic activity was observed in fraction 15, as shown in Figure 8. When azocasein was used as the substrate, this fraction exhibited a 1.22-fold increase relative to the crude extract, suggesting an apparent enrichment of active proteases in that elution range, although this may rather reflect the removal of interfering substances affecting azocasein measurement. In contrast, when casein was used as the substrate, fraction 15 also showed the highest activity within the profile, but without apparent gain compared to the crude extract, consistent with the results observed for BC and P2. This discrepancy between substrates is expected, since azocasein provides a more sensitive detection of a broad spectrum of proteases through the release of a quantifiable chromophore, whereas the casein method primarily measures tyrosine and soluble peptide release [46,47].

Previous studies on the purification of proteases from *P. aeruginosa* describe multistep purification schemes, including salt precipitation, ion-exchange chromatography, and gel filtration (size-exclusion) chromatography. In the latter step, studies reported that using Sephadex G-150 and G-200 columns achieved purification factors of 4.1 and 2.7, respectively. In other words, the specific activity of the enzyme increased considerably compared to the crude extract. [48,49].

#### 2.3.4. Molecular Size Determination

**Figure 9:**
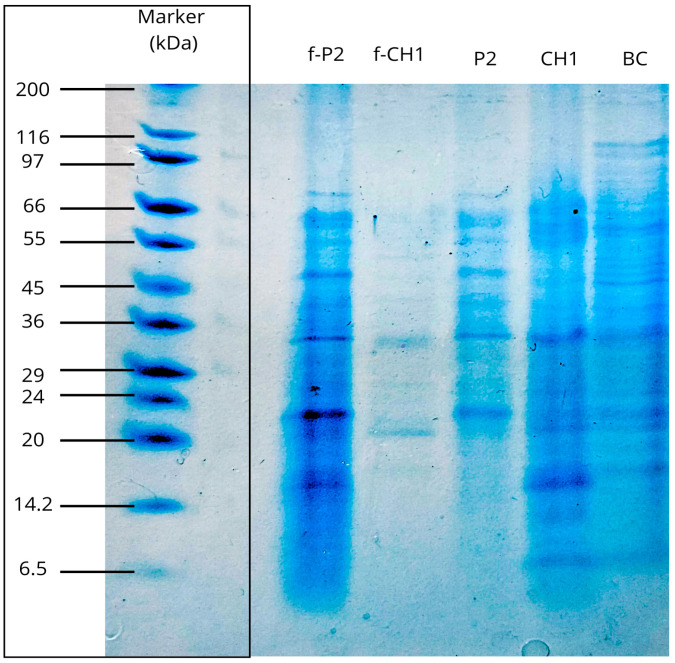
SDS-PAGE profile of semipurified fractions obtained by Sephadex G-100 (f-P2 and f-CH1) and crude enzyme extracts from P2 (*Bacillus paramycoides*), CH1 (*Pseudomonas aeruginosa*) and BC (*Enterobacter cloacae*).

SDS-PAGE analysis (Figure 9) revealed a differential band pattern between approximately 15 and 116 kDa when comparing crude extracts and semipurified fractions of proteases produced by strains BC (*Enterobacter cloacae*), P2 (*Bacillus paramycoides*), and CH1 (*Pseudomonas aeruginosa*).

The crude extract of BC (*Enterobacter cloacae*), shown in the fifth lane, displayed a faint and diffuse signal predominantly in the upper region of the gel (≥~60 kDa), suggesting a low relative abundance of the target proteases, possible aggregation, or comigration with high-molecular-weight proteins. Previous studies indicate that *Enterobacter* proteases typically exhibit molecular weights between 46 and 56 kDa, characteristic of metalloproteases that are of biomedical relevance for understanding bacterial infection control [50].

The semipurified fraction corresponding to the protease from *Bacillus paramycoides* (f-P2) displayed five predominant bands at approximately 15, 22, 35, 50, and 66 kDa, whereas the crude enzymatic extract (P2) showed a heterogeneous pattern with multiple bands between ~15 and 66 kDa. This behavior indicates a reduction in protein complexity and relative enrichment after gel filtration; however, the persistence of several bands in f-P2 suggests coelution of other proteins or isoforms.

The observed mass distribution is consistent with the typical range for alkaline proteases from *Bacillus* (e.g., serine proteases of the subtilisin type ~30–35 kDa and metalloproteases ~50–70 kDa) [51]. In this context, a detergent-compatible serine protease produced by *Bacillus safensis* strain PRN1, with an approximate molecular weight of 33 kDa, has been successfully purified using different analytical techniques, supporting the prevalence of enzymes within this size range among industrially relevant *Bacillus* proteases [52]. Furthermore, several studies have reported that *B. paramycoides* produces alkaline proteases (pH 9–10; temperature 40–45 °C) with potential industrial applications, particularly as additives in eco-friendly detergents [41,53]. Likewise, a collagenase of approximately 60 kDa has been documented from *B. paramycoides* isolated from slaughterhouse soil, showing potential use in bioremediation [54].

The semipurified fraction of *Pseudomonas aeruginosa* (f-CH1) exhibited two predominant bands near 20 and 35 kDa, whereas the crude enzymatic extract (CH1) showed multiple bands between ~6.5 and 66 kDa, indicating protein enrichment and, therefore, effective semipurification after the chromatographic step. The ~35 kDa band presumably corresponds to a metalloprotease elastase known as pseudolysin, listed in the BRENDA database under the accession number EC 3.4.24.26. This enzyme was reported to be secreted by *P. aeruginosa* isolated from cyclohexane-contaminated soil, conferring solvent stability and higher specific activity, which reinforces its biotechnological potential [55]. The ~20 kDa band may correspond to staphylolysin/LasA (EC 3.4.24.B16), which is used in biomedical research to study protein degradation mechanisms, bacterial invasion processes, and to develop metalloprotease inhibitors as potential therapeutic agents [56].

Regarding the bioinformatic research, no specific entries documenting extracellular proteases for *Bacillus paramycoides* or *Enterobacter cloacae* were found in the BRENDA enzyme database. The extracellular proteases of these strains may not have been functionally characterized or deposited in public databases, may exist under non-standardized nomenclature, or may exhibit significant structural differences compared to enzymes reported in other genera.

## 3. Materials and Methods

### 3.1. Bacterial Bioprospecting

Bacterial bioprospecting was conducted under an exploratory, discovery-oriented approach, with emphasis on the regional ecological gradient spanning Ecuador’s regions. The country offers an ideal setting for such research, as it comprises three well-defined climatic regions: Coast, Highlands, and Amazon region. For this study, one representative city was selected from each region. Quito (Pichincha), located at 2850 m a.s.l. in the Highlands, has a temperate climate with temperatures between 9 °C and 21 °C. Bucay (Guayas), situated in the Coast at 320 m a.s.l., is characterized by a warm, humid climate with temperatures ranging from 16 °C to 25 °C. Puyo (Pastaza), in the Amazon region at 950 m a.s.l., exhibits a tropical rainforest climate with temperatures between 18 °C and 28 °C [57,58].

These environmental variations are associated with greater heterogeneity of microbial communities within the isolation matrices at the time of sampling, thereby increasing the likelihood of isolating proteolytic bacteria that exhibit stability and activity under operational parameters of industrial interest.

#### 3.1.1. Sampling Strategy and Sample Collection

Samples were collected in the cities of Quito, Bucay and Puyo. In Quito, sampling was carried out in a local market on pork (*Sus scrofa domesticus*) and beef (*Bos taurus*), and in trout ponds (*Oncorhynchus mykiss*), where surface water and biofilm were sampled. In Bucay (*Coastal region*), sampling was performed in a local market on common snook (*Centropomus undecimalis*) and pork, and in the Chimbo River, where surface water and biofilm were collected. In Puyo (Amazon region), samples were obtained in the city market from South American palm weevil (*Rhynchophorus palmarum*), pork (*Sus scrofa domesticus*), tilapia (*Oreochromis niloticus*) and from the water of tilapia storage tanks.

At each sampling site, matrix handling and primary inoculation were performed in situ. For meat, fish and chontacuro samples, small portions from the inner part of each matrix were aseptically taken with sterile instruments and directly smeared onto the surface of modified gelatin agar plates, followed by streaking. For surface water, a sterile loop was immersed in the upper water layer and immediately used to inoculate the plates. In the case of biofilms, the agar surface of the modified gelatin plates was gently pressed directly against the biofilm (contact-plate method) to transfer adherent cells. All inoculated plates were then transported to the laboratory in insulated containers and incubated under the conditions described below.

#### 3.1.2. Bacterial Isolation

Aerobic bacteria were isolated on modified gelatin agar (6.67% bovine gelatin, 0.5% natamycin, and 1.2% agar). The bovine gelatin used in the medium was a commercial product (260 Bloom, USP grade) purchased from La Casa de los Químicos (Quito, Ecuador). The medium was sterilized, dispensed into sterile Petri dishes, and stored at 4 °C until use [8,59]. At each sampling site, inoculation was performed in situ by direct smear of the samples followed by duplicate streaking. The plates were then transported to the Microbiology Laboratory of the Department of Biotechnology and Food Sciences (DECAB), where they were incubated at 37 °C for 24 h. Morphologically distinct colonies were purified by successive subculturing on the same medium. Pure cultures were preserved in Brain Heart Infusion (BHI) broth with 15% glycerol and stored at −80 °C. Uninoculated negative controls were included at all stages of the isolation process.

### 3.2. Bacterial Identification

#### 3.2.1. Biochemical Tests

Biochemical and morphological tests recommended in *Bergey’s Manual of Systematic Bacteriology* [60] were performed for the identification of the isolated strains. Gram staining was conducted using the conventional crystal violet–iodine method, and cell morphology and Gram reaction were examined under oil immersion microscopy [61]. Endospore formation was evaluated by spore staining using the Schaeffer–Fulton method, with malachite green as the primary stain and safranin as the counterstain [62]. Catalase activity was determined by adding 3% (*v*/*v*) hydrogen peroxide to fresh bacterial colonies and observing immediate oxygen bubble formation [61]. Urease activity was assessed on urea agar slants by monitoring the development of a pink coloration due to the phenol red indicator after incubation at 37 °C [63].

Carbohydrate fermentation was evaluated using basal media supplemented with specific carbohydrates and a pH indicator, with acid production evidenced by a color change and gas formation recorded when applicable. Hydrogen sulfide (H_2_S) production was determined using sulfide-indicator media, with positive results indicated by black precipitate formation [64]. Motility was assessed using semi-solid agar stab cultures, where diffuse growth away from the inoculation line indicated motile strains [65]. Lactose utilization was evaluated using lactose-containing differential media based on color changes associated with acid production [26]. Gelatin hydrolysis was tested by inoculating gelatin-containing media and assessing liquefaction after incubation and refrigeration, indicating proteolytic activity [27].

#### 3.2.2. Molecular Identification

Genomic DNA was extracted by alkaline lysis by resuspending a bacterial colony in 60 µL of 50 mM NaOH and incubating at 100 °C for 5 min, resulting in cell envelope disruption and release of DNA into solution [66].

The 16S rRNA gene was amplified by PCR. A 10 µL reaction mixture was prepared containing 5 µL GoTaq^®^ Master Mix (2X) (Promega, Madison, WI, USA), 0.12 µL each of universal primers 27F and 1492R (10 µM), 2.88 µL nuclease-free water, and 2 µL of extracted DNA, following the manufacturer’s protocol.

Subsequently, samples were centrifuged and amplified in a thermal cycler under the following conditions: initial denaturation at 95 °C for 5 min; 35 cycles of 95 °C for 40 s, 56 °C for 40 s, and 72 °C for 1 min 40 s; and a final extension at 72 °C for 5 min. Amplicon quality was verified by electrophoresis on a 1% agarose gel in 1× TAE buffer using ECO-STAIN as the intercalating dye. Amplicons and a 100-bp DNA ladder (BioBasic, ready-to-use) were loaded, electrophoresed at 160 V for 30 min, and visualized under ultraviolet light [67,68].

Amplicons were sent to Macrogen Inc. (Seoul, Republic of Korea) for Sanger sequencing, and the resulting sequences were compared against NCBI databases using BLAST to determine the taxonomic identity of the isolates.

### 3.3. Evaluation of the Proteolytic Capacity of the Identified Strains

#### 3.3.1. Production of Proteases

Each bacterial strain, including a *Bacillus subtilis* strain (positive control for protease production), was inoculated (100 µL), in triplicate, into Erlenmeyer flasks containing 50 mL of previously sterilized culture medium composed of 0.1 M phosphate buffer (pH 7), 0.5% sodium chloride, 0.20% dextrose, and 1% soybean paste [69].

The culture flasks were placed in a shaker incubator at 37 °C, 120 rpm, for a minimum of 72 h.

#### 3.3.2. Enzyme Precipitation

After the production period, the enzyme was precipitated by adding ammonium sulfate (40%) to the culture media with constant stirring in an ice bath for 1 h. The samples were then refrigerated for an additional hour and centrifuged at 10,000× *g* for 10 min. The supernatants were discarded, and the resulting pellets were reconstituted in 0.1 M Tris-HCl buffer (pH 8.0) [70].

#### 3.3.3. Quantification of Proteolytic Activity

Proteolytic activity of the extracts was quantified in triplicate using two complementary assays: the casein assay, which measures the release of peptides and aromatic amino acids, and the azocasein assay, which evaluates colored peptide fragments linked to azo groups [46,47].

The first method used azocasein as the substrate. For the sample tube, 200 µL of 0.1 M Tris-HCl buffer (pH 8), 200 µL of the enzyme extract, and 200 µL of 1% azocasein (dissolved in 0.02 M Tris-HCl, pH 8) were added. For the blank, the same reagents were used in a separate tube, replacing the enzyme extract with 200 µL of distilled water, and 1 mL of 10% trichloroacetic acid (TCA) was added immediately. Both tubes were then incubated at 37 °C for 30 min; after incubation, 10% TCA was added to the sample. The tubes were centrifuged at 10,000× *g* for 15 min, and 400 µL of 1.8 N NaOH was added to the supernatant. Absorbance was measured at 420 nm using a UV–Vis spectrophotometer [71].

For the second method, casein was used as the substrate, following the Anson method with some modifications [72]. For the sample tube, 100 µL of the enzyme extract were added, followed by 1.1 mL of 1% casein (dissolved in 50 mM phosphate buffer, pH 7). In parallel, a blank was prepared in a separate tube by adding 100 µL of distilled water, 1.1 mL of casein, and 1.8 mL of 10% trichloroacetic acid (TCA). Both preparations were incubated at 37 °C for 20 min. After incubation, the reaction in the sample was stopped by adding 1.8 mL of 10% TCA. The tubes were then centrifuged at 4500× *g* for 20 min, and the resulting supernatant was collected to determine its optical density at 280 nm using a UV–Vis spectrophotometer [72].

For both assays, one unit of proteolytic activity (U) was defined as the amount of enzyme that produced an increase of 0.1 in absorbance under the specified conditions, and enzymatic activity (EA) was expressed as U/mL [69].

#### 3.3.4. Kinetics of Enzyme Production and Bacterial Growth

To determine the time of maximum enzymatic production of the strain selected as the highest protease producer, 21 Erlenmeyer flasks were prepared, each containing 50 mL of previously sterilized culture medium. The medium consisted of phosphate buffer (0.1 M, pH 7), 0.5% sodium chloride, 0.2% dextrose, and 1% soybean paste [69,73]. The flasks were then incubated with agitation at 37 °C and 120 rpm for a total of 7 days.

Every 24 h, three flasks were removed from the incubator to determine the viable bacterial count by surface plating using serial dilutions [74] and to measure the proteolytic activity as described in Section 2.3.3.

#### 3.3.5. Partial Purification of the Enzyme

The crude extract collected on the day of maximum enzymatic production was subjected to partial purification by gel filtration chromatography using a column 28 cm in height and 7 mm in diameter, packed with Sephadex G-100 resin previously hydrated for 24 h in phosphate buffer (0.1 M, pH 7). A 1 mL aliquot of the enzyme extract was injected, and 0.5 mL fractions were collected. Enzymatic activity in each fraction was quantified as previously described to determine the major activity fraction [75].

The major enzymatic fractions and the crude enzyme extracts were lyophilized and stored at 4 °C for preservation.

#### 3.3.6. Molecular Size Determination

The molecular size of the fraction with the highest proteolytic activity and of the crude enzyme extracts was determined by vertical polyacrylamide gel electrophoresis (SDS-PAGE) using the Mini-PROTEAN II system (Bio-Rad Laboratories, Hercules, CA, USA). The procedure followed the method described by Schägger and von Jagow [76], which allows for the separation of proteins ranging from 1 to 100 kDa by using tricine to improve the resolution of low-molecular-weight proteins without the need for urea.

Separating gels (10% T, 3% C) and stacking gels (4% T, 3% C) were prepared using ammonium persulfate (APS, 10%) and TEMED as polymerization catalysts. After polymerization, gels were assembled in the electrophoresis unit, and 10 µL of molecular weight marker (6.5–200 kDa; Sigma-Aldrich, St. Louis, MO, USA) and 10 µL of enzyme extract denatured at 90 °C for 10 min were loaded per well. Electrophoresis was performed at 35 V until the samples entered the separating gel, followed by 60 V until migration was completed.

Subsequently, the gel was immersed in staining solution for 24 h and then in destaining solution for 2 h before visualization. The activity bands observed in the gels were associated with the corresponding producing strains and based on their apparent molecular mass and substrate profile, were used to propose the identity of the proteases, which was cross-referenced with the BRENDA enzyme database [77].

#### 3.3.7. Statistical Analysis

All experiments were performed in triplicate, and the results were expressed as mean ± standard deviation (SD). Normality and homoscedasticity were evaluated using the Shapiro–Wilk and Levene tests (*p* < 0.05). Significant differences among treatments were determined by one-way ANOVA followed by Duncan’s multiple range post hoc test (α = 0.05). All statistical analyses were carried out using Statgraphics Centurion^®^ XVI.I software (version 16.1.03).

## 4. Conclusions

In the present study, 34 bacterial strains were isolated and identified from meat matrices and water sources collected across three distinct biogeographical regions of Ecuador (Andean, Coastal, and Amazon). These strains were taxonomically assigned to the orders *Enterobacterales*, *Pseudomonadales*, and *Bacillales* through biochemical characterization and 16S rRNA gene analysis, highlighting the influence of regional environmental conditions on the diversity of bacterial communities with proteolytic potential.

The evaluation of proteolytic activity demonstrated that ecological origin and substrate availability in the isolation matrix play a relevant role in the selection and expression of this phenotype. Among the isolates, *Enterobacter cloacae* (BC, pork, Quito), *Bacillus paramycoides* (P2, snook, Bucay), and *Pseudomonas aeruginosa* (CH1, chontacuro, Puyo) exhibited the highest proteolytic activity within their respective regions.

Enzymatic production kinetics revealed distinct activity profiles among these strains. While *E. cloacae* and *B. paramycoides* reached maximum proteolytic activity on day four, followed by a decline, *P. aeruginosa* (CH1) showed early enzyme production, peaking on day two and maintaining relatively stable activity over time. This rapid onset and sustained activity profile indicates a production behavior that is compatible with industrial protease processes requiring efficiency and operational stability.

Partial purification by gel filtration chromatography (Sephadex G-100) successfully concentrated the proteolytic enzymes, resulting in fractions with significantly enhanced activity. SDS-PAGE analysis of semipurified extracts from *B. paramycoides* and *P. aeruginosa* revealed protein bands with molecular weights consistent with reported serine proteases, metalloproteases, and elastases, supporting the biotechnological relevance of the enzymes produced.

Overall, the results demonstrate that at least one isolate, *Pseudomonas aeruginosa* (CH1), exhibits proteolytic activity levels, production kinetics, and stability profiles that are acceptable for consideration as a promising bacterial producer for industrial enzyme production at the laboratory scale. Nevertheless, further strain selection, optimization of culture conditions, and detailed evaluation under industrially relevant conditions (pH, temperature, salinity, and scale-up) are required before definitive industrial application.

## Figures and Tables

**Table 1:** Origin of isolated bacteria by city and isolation matrix.

City—Region	Sampling Site—Coordinates	Matrix	Bacterial Isolation Identifier
Quito (Andean region)	“La Kennedy” Market (0°08′21.3″ S 78°28′40.7″ W)	Pork meat	BC, TC
		Beef	BR, TRA, TRB
	“La Ponderosa” Tourist Center (0°22′42.8″ S 78°23′22.4″ W)	Surface water	P2A
		Biofilm	P1B, P2B
Bucay (Coastal region)	Municipal Market (2°11′59.1″ S, 79°08′18.0″W)	Common snook	P1, P2, P3, P5
		Pork meat	C1, C2, C3, C4, C5
	Chimbo River (2°12′10.7″ S, 79°08′21.0″ W)	Surface water	RA1, RA2, RA3
		Biofilm	RB1, RB2, RB3, RB4
Puyo (Amazon region)	“Mariscal” Market (1°29′13.5″ S 77°59′42.5″ W)	Chontacuro	CH1, CH2, CH3
		Pork meat	CA1, CA2
		Tilapia	PE1, PE2
		Surface water	AP1, AP2, AP3

**Table 2:** Taxonomic Assignment of Isolated Bacteria Based on BLASTn Alignment of 16S rRNA Gene Sequences.

City—Region	Identifier	Organism (16S BLAST)	Size (pb)	Identity (%)	GenBank Accession Number
Quito—Andean region	BC	*Enterobacter cloacae*	757	99.87	NR_118568.1
	TC	*Citrobacter pasteurii*	758	99.60	OR660260.1
	BR	*Citrobacter freundii*	851	99.41	NR_113340.1
	TRA	*Kocuria tytonicola*	1001	99.50	NR_179862.1
	TRB	*Citrobacter pasteurii*	910	99.89	OR660260.1
	P1B	*Escherichia fergusonii*	754	100	CP083638.1
	P2A	*Enterobacter cloacae*	941	99.68	NR_118568.1
	P2B	*Serratia fonticola*	886	99.89	CP011254.1
Bucay—Costal region	P1	*Bacillus paramycoides*	972	100	OQ366607.1
	P2	*Bacillus paramycoides*	880	100	OQ366607.1
	P3	*Pseudomonas aeruginosa*	565	99.82	LN831024.1
	P5	*Citrobacter braakii*	668	98.84	KM515967.1
	C1	*Klebsiella quasivariicola*	840	99.17	CP022823.1
	C2	*Serratia marcescens*	890	99.89	PP095668.1
	C3	*Pseudomonas paraeruginosa*	881	99.89	ON359917.1
	C4	*Pseudomonas aeruginosa*	992	99.90	NR_113599.1
	C5	*Serratia marcescens*	862	99.77	CP071236.1
	RA1	*Klebsiella pneumoniae*	882	99.89	OR002049.1
	RA2	*Escherichia coli*	783	100	CP033092.2
	RA3	*Klebsiella pneumoniae*	916	99.78	OR002049.1
	RB1	*Klebsiella quasipneumoniae*	906	99.78	CP084876.1
	RB2	*Citrobacter amalonaticus*	917	99.67	AP024585.1
	RB3	*Klebsiella quasipneumoniae*	811	99.75	CP084787.1
	RB4	*Klebsiella quasivariicola*	915	99.34	CP022823.1
Puyo—Amazon region	CH1	*Pseudomonas aeruginosa*	1002	99.70	MK796437.1
	CH2	*Serratia marcescens*	723	99.86	PP095668.1
	CH3	*Klebsiella variicola*	870	99.89	CP084765.1
	CA1	*Klebsiella variicola*	577	100	NR_025635.1
	CA2	*Shigella flexneri*	908	100	OQ618959.1
	PE1	*Stenotrophomonas acidaminiphila*	879	99.77	NR_025104.1
	PE2	*Enterobacter asburiae*	704	99.15	CP011863.1
	AP1	*Klebsiella quasivariicola*	894	99.55	CP022823.1
	AP2	*Acinetobacter soli*	988	99.80	NR_044454.1
	AP3	*Klebsiella variicola*	926	99.68	NR_025635.1

## Data Availability

The original contributions presented in this study are included in this article; further inquiries can be directed to the corresponding author.

## Abbreviations

GLU	glucose
LAC	lactose
SUC	sucrose
NG	no growth

## Appendix A

**Table A1:** Biochemical test results of isolated bacterial strains (+/− indicates a non-conclusive result in the biochemical tests).

City—Region	Identifier	Gram	Spores	Catalase	Urease	GLU	GLU/LAC/SUC	H_2_S	Gas	Mcconkey—LAC	Motility	Gelatin
Quito—Andean region	BC	−	−	+	−	+	+	−	+	+	+	−
	TC	−	−	±	−	+	+	+	+	+	−	−
	BR	−	−	±	+	+	+	+	−	+	+	−
	TRA	+	−	+	+	−	−	−	−	NG	+	−
	TRB	−	−	+	+	+	−	+	+	+	+	−
	P1B	−	−	+	−	+	+	−	+	+	+	−
	P2A	−	−	+	+	+	+	−	+	+	−	−
	P2B	−	−	+	−	+	−	−	−	+	+	−
Bucay—Coastal region	P1	+	+	+	+	+	−	−	−	NG	+	+
	P2	+	+	+	−	+	+	−	−	NG	+	+
	P3	−	−	+	−	−	−	−	−	+	−	+
	P5	−	−	+	−	+	+	+	+	+	+	−
	C1	−	−	+	+	+	+	−	+	+	+	−
	C2	−	−	+	+	+	+	−	−	−	+	−
	C3	−	−	+	+	−	−	−	−	+	−	+
	C4	−	−	+	+	−	−	−	−	+	−	−
	C5	−	−	+	−	+	+	−	−	−	−	+
	RA1	−	−	+	+	+	+	−	+	+	−	−
	RA2	−	−	+	+	+	+	+	−	+	−	−
	RA3	−	−	+	+	+	+	−	+	+	−	−
	RB1	−	−	+	+	+	+	−	+	−	−	−
	RB2	−	−	+	+	+	+	−	−	+	+	−
	RB3	−	−	+	+	+	+	−	±	+	−	−
	RB4	-	−	+	+	+	+	−	+	+	−	−
Puyo—Amazon region	CH1	−	−	+	−	−	−	−	−	+	+	+
	CH2	−	−	+	+	+	−	−	−	−	−	+
	CH3	−	−	+	+	+	+	−	+	+	−	−
	CA1	−	−	+	+	+	+	−	+	+	−	−
	CA2	−	−	+	−	+	+	−	+	+	−	−
	PE1	−	−	+	−	−	−	−	−	−	+	−
	PE2	−	−	+	−	+	−	−	+	+	+	−
	AP1	−	−	+	−	+	+	−	+	+	+	−
	AP2	−	−	+	+	−	−	−	−	+	+	−
	AP3	−	−	+	+	+	+	−	+	+	+	−
